# Supplementary material for: Stearoyl-CoA Desaturases1 Accelerates Non-Small Cell Lung Cancer Metastasis by Promoting Aromatase Expression to Improve Estrogen Synthesis
Source: Int J Mol Sci. 2023 Apr 6;24(7):6826. doi: 10.3390/ijms24076826 (PMC10095487; doi:10.3390/ijms24076826)
Supplement: Supplementary file 1 [file ijms-24-06826-s001.zip › ijms-2255609-supplementary.pdf]

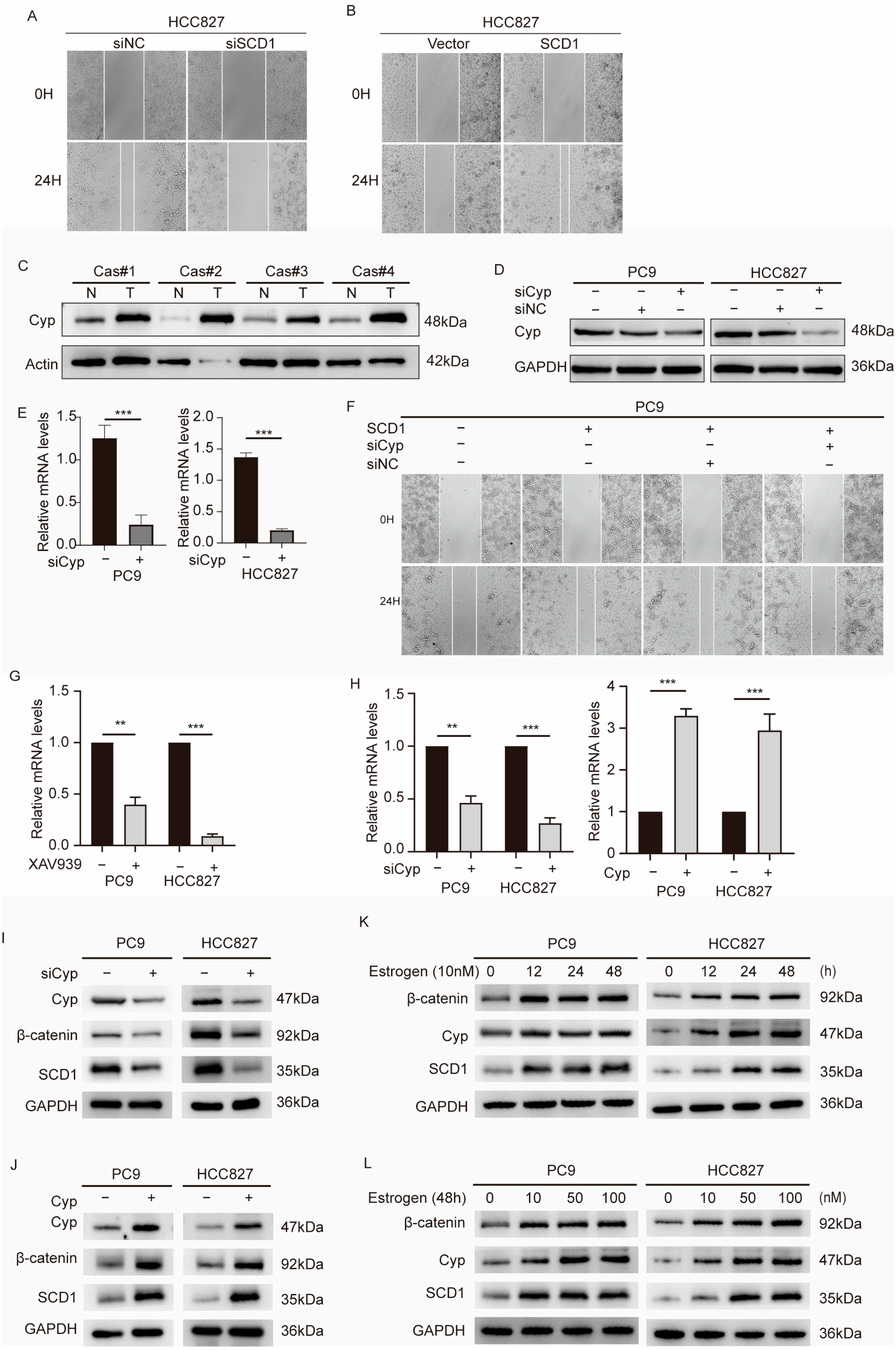

Supplementary Figure S1. A, B The migration ability of transfected PC9 evaluated by wound healing assays. **C** The CYP19A1 protein expression levels in lung cancer tissues and

corresponding pericancerous tissues as measured by Western blotting. **D, E** The protein and mRNA ($n = 3$) levels were verified by Western blotting and qRT-PCR in PC9 and HCC827 cells with transient CYP19A1 knockdown. **F** The migration ability of transfected PC9 cells was evaluated by wound healing assays. **G** The CYP19A1 mRNA level in PC9 and HCC827 cells treated with XAV939 for 48 h. **H** The SCD1 mRNA levels in CYP19A1 knockdown (left) and overexpression (right) cells. The SCD1 protein levels in CYP19A1 **I** knockdown and **J** overexpression cells. **K** The protein levels of β -catenin, CYP19A1, and SCD1 when exposed to estrogen at a concentration of 10 nM for 12, 24, and 48 hours in PC9 and HCC827 cell lines. **L** The β-catenin, CYP19A1, and SCD1 protein levels when exposed to 0, 10, and 50, 100 nM estrogen for 48 hours. **$p < 0.01$; ***$p < 0.001$. Error bars indicate mean ± SD. Cyp: CYP19A1.

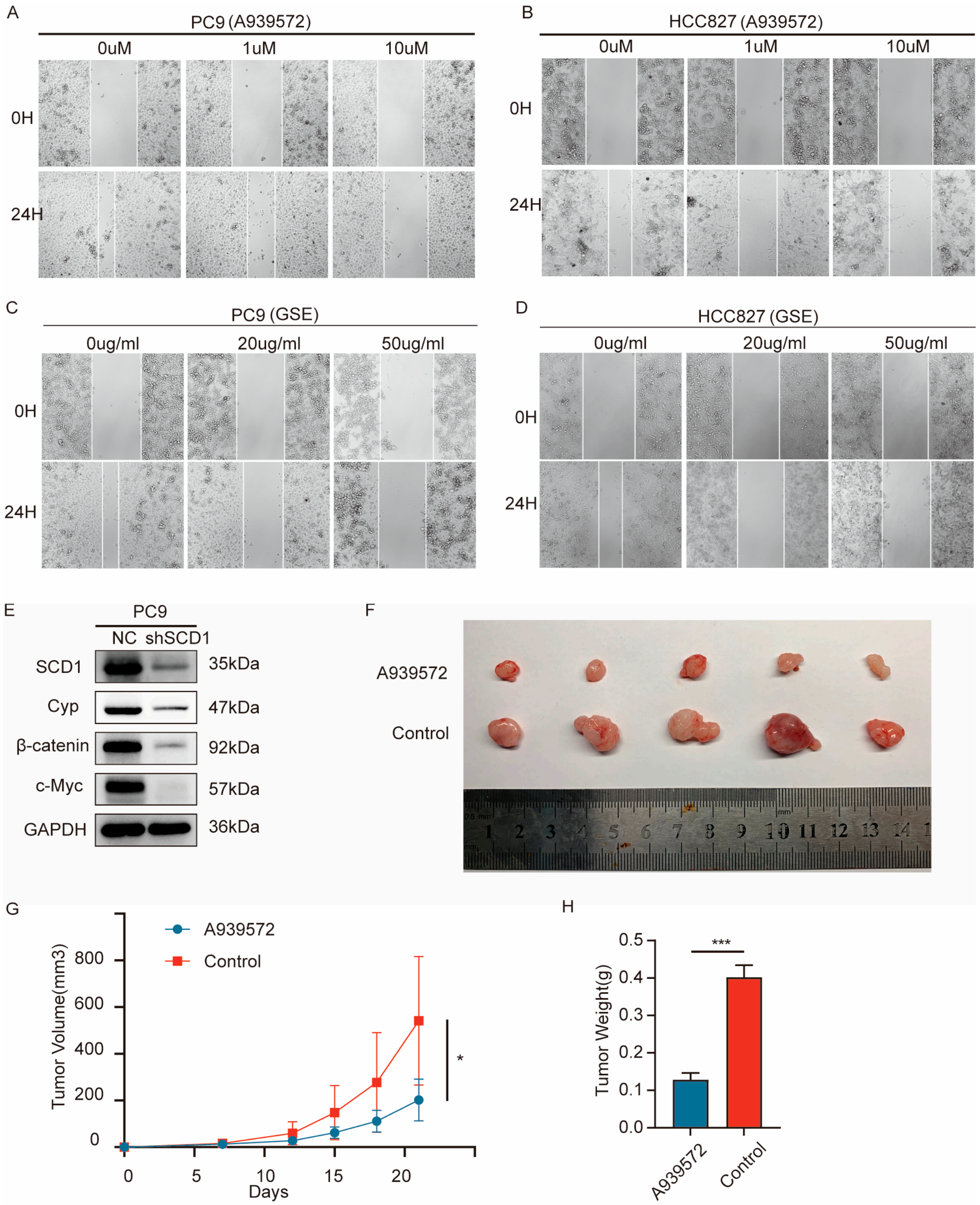

Supplementary Figure S2. The migration ability of **A** PC9 and **B** HCC827 cells treated with A939572 was evaluated by wound healing assays. The migration ability of **C** PC9 and **D** HCC827 cells treated with GSE was evaluated by wound healing assays. **E** The protein levels of SCD1, Cyp, β-catenin, and c-Myc with stable knockdown of SCD1. PC9 cells were used for xenograft tumor, **F** tumor size, **G** tumor growth curve, **H** tumor weight were displayed. A939572 (50mg/kg, p.o., q3d) treatment was started on the fourth day, total 21 days. ***$p < 0.001$. Error bars indicate mean ± SD. Cyp: CYP19A1.
